# Draft Genome Sequence of *Rhodococcus rhodnii* Strain LMG5362, a Symbiont of *Rhodnius prolixus* (Hemiptera, Reduviidae, Triatominae), the Principle Vector of *Trypanosoma cruzi*

**DOI:** 10.1128/genomeA.00329-13

**Published:** 2013-06-20

**Authors:** Justin A. Pachebat, Geertje van Keulen, Miranda M. A. Whitten, Susan Girdwood, Ricardo Del Sol, Paul J. Dyson, Paul D. Facey

**Affiliations:** Institute of Life Science, College of Medicine, Swansea University, Singleton Park, Swansea, United Kingdomb; Institute of Biological, Environmental and Rural Sciences, Aberystwyth University, Penglais, Aberystwyth, Ceredigion, United Kingdoma

## Abstract

We report the 4,385,577-bp high-quality draft assembly of the bacterial symbiont *Rhodococcus rhodnii* strain LMG5362, isolated from the gut of *Rhodnius prolixus* (Hemiptera, Reduviidae, Triatominae), the principle vector of the protozoan *Trypanosoma cruzi*, the etiological agent of Chagas disease. This sequence might provide useful information for subsequent studies of the symbiotic relationship between *Rd. prolixus* and *Rc. rhodnii*, while also providing a starting point for the development of biotechnological applications for the control of *Rd. prolixus*.

## GENOME ANNOUNCEMENT

The genus *Rhodococcus* comprises metabolically diverse nocardioform high-G+C-content actinomycetes that have been isolated from a wide variety of sources (1). The genus includes *Rhodococcus rhodnii*, an endosymbiont of the triatomine bug *Rhodnius prolixus*. *Rd. prolixus* has a neotropical distribution, where it is the principle vector of the stercorarian protozoan parasite *Trypanosoma cruzi*, the etiological agent of Chagas disease (South American trypanosomiasis). This neglected tropical disease is associated with poverty, lacks both a vaccine and an effective drug treatment for its chronic phase, and causes >12,000 mortalities annually through damage to the heart and central nervous system of humans (2, 3). Chagas disease also carries a disproportionately high economic burden estimated at $7 billion per year (2). *Rd. prolixus* is an important parasite of humans and domestic animals and is an obligate blood feeder throughout its hemimetabolous life cycle (4). These insects appear to be dependent on a symbiotic relationship with *Rc. rhodnii* to supplement their nutrient-poor diet, in particular with B vitamins (5). Indeed, *Rc. rhodnii* is passed from adult to offspring by coprophagy, with aposymbiotic *Rd. prolixus* dying prematurely during nymphal development (5).

*Rc. rhodnii* LMG5362, isolated from the hind gut of *Rd. prolixus*, was obtained from the Belgian Co-ordinated Collections of Micro-organisms (BCCM). A single colony of *Rc. rhodnii* was grown in tryptic soy broth (TSB) at 28°C, with 250 rpm shaking, for 4 days. Total genomic DNA was isolated using the Fast DNA spin kit for soil (MP Biomedicals, Santa Ana, CA) and quantified using a Quant-iT BR DNA assay (Invitrogen). Five hundred nanograms of genomic DNA was fragmented by nebulization and used to make a 454 Lib-L sequencing library, which was shotgun sequenced using a 454/Roche GS-FLX Titanium platform (454 Life Sciences, Branford, CT). This generated 601,588 reads, with an average length of 437 bp, and total of 262,813,142 bp. Reads were assembled into 140 contigs using the GS *de novo* assembler (version 2.3) with default parameters, aligning 99.45% of reads, with a mean read depth of 56.0. The largest contig size is 223,927 nucleotides (nt). Annotation was performed using xBASE2 (6). Briefly, open reading frames (ORFs), tRNA genes, and rRNA genes were identified with Glimmer (7), tRNAscan-SE (4) and RNAmmer (8), respectively. Fourteen contigs contained no identifiable ORFs. The estimated genome size of LM5362 is 4,385,577 bp, with a G+C content of 69.71 mol%. The draft genome contains 4,418 putative ORFs (average length, 900 bp), 50 tRNA genes, and 3 rRNA genes. Furthermore, the genome encodes >30 genes or gene clusters for natural product biosynthesis. These include at least seven polyketide and/or fatty acid synthases, eight nonribosomal peptide synthases, of which two have homology to isopenicillin N synthases, and one cluster for phytoene or carotenoid biosynthesis. Additionally, vitamin B biosynthesis genes could be identified, including genes for the biosynthesis of thiamine (B_1_), riboflavin (B_2_), niacin (B_3_), pantothenate (B_5_), pyridoxal (B_6_), biotin (B_7_), tetrahydrofolate (B_9_), and cobalamin (B_12_).

### Nucleotide sequence accession numbers.

This Whole-Genome Shotgun project has been deposited at DDBJ/EMBL/GenBank under the accession no. APMY00000000. The version described in this paper is the first version, accession no. APMY01000000.
